# Application of a Ridden Horse Pain Ethogram and Its Relationship with Gait in a Convenience Sample of 60 Riding Horses

**DOI:** 10.3390/ani10061044

**Published:** 2020-06-17

**Authors:** Sue Dyson, Danica Pollard

**Affiliations:** 1 Independent Consultant, The Cottage, Church Road, Market Weston, Diss IP22 2NX, UK; 2 Epidemiology and Disease Surveillance Department, Centre for Preventive Medicine, Animal Health Trust, Newmarket, Suffolk CB8 7UU, UK; drdee.pollard@gmail.com

**Keywords:** lameness, musculoskeletal pain, canter, behaviour, dressage, riding school, saddle-fit, rider skill, thoracolumbar pain

## Abstract

**Simple Summary:**

Horse owners are poor at recognising lameness which may compromise equine welfare. A Ridden Horse Pain Ethogram, comprising 24 behaviours with specific definitions, was developed to facilitate identification of musculoskeletal pain. Previous studies demonstrated that the presence of ≥8/24 behaviours is likely to reflect musculoskeletal pain. The aim of this study was to further test the Ridden Horse Pain Ethogram by applying it to a convenience sample (*n* = 60) of sports horses and riding school horses in regular work and assumed by their owners to be working comfortably. All horses performed a purpose-designed dressage-type test of 8.5 min duration in walk, trot and canter, with their normal rider. The presence of increased back muscle tension or pain, poor saddle fit, gait abnormalities and rider skill were assessed by independent experts. The Ridden Horse Pain Ethogram was applied retrospectively, by a trained analyst, to video recordings which had been acquired in a standardised fashion. There was a significant association between the Ridden Horse Pain Ethogram score and lameness. Lame horses had higher scores than non-lame horses. Education of riders about behaviours which may reflect pain in ridden horses could allow the earlier identification of lame horses, whose welfare may be improved by accurate diagnosis and treatment.

**Abstract:**

A Ridden Horse Pain Ethogram (RHpE) comprising 24 behaviours has been developed to facilitate the identification of musculoskeletal pain. The aim was to further test the RHpE by its application to a convenience sample (*n* = 60) of sports horses and riding school horses in regular work and assumed by their owners to be working comfortably. All horses performed a purpose-designed dressage-type test of 8.5 min duration in walk, trot and canter, with their normal rider. The RHpE was applied retrospectively to video recordings acquired in a standardised fashion. Seventy-three percent of horses were lame (≤ grade 2/8) on one or more limbs; 47% had gait abnormalities in canter. Ridden Horse Pain Ethogram scores ranged from 3 to 16/24 (median 9); rider skill score ranged from 2.5 to 8/10 (median 4.75). The effect of horse age, breed, sex, work-discipline, epaxial muscle hypertonicity or pain, an ill-fitting saddle, rider skill score, the presence of lameness or gait abnormalities in canter on the RHpE score was assessed using Poisson regression. Two variables were retained in the final multivariable analysis, rider skill score as a continuous variable (*p* < 0.001), and lameness (*p* = 0.008). A RHpE score ≥8 was a good indicator of the presence of musculoskeletal pain.

## 1. Introduction

In 2012 it was estimated that there were at least 840,000 horses in Great Britain [1]. There is limited information available about the frequency of occurrence of lameness and other sources of musculoskeletal pain in sports horses and riding school horses. In the United Kingdom’s Blue Cross National Equine Health survey for 2018, lameness accounted for 23% of all disease syndromes reported for 13,873 horses [2]. However, there is evidence that owners and trainers are poor at lameness recognition; in a United Kingdom survey of 506 sports horses in full work and presumed to be non-lame, 47% were lame or had other pain-related gait abnormalities [3]. In a Swedish study of sports horses in full work and functioning normally, 53% of 201 horses showed measurable asymmetry of gait when trotted in hand [4].

In order to facilitate the recognition of musculoskeletal pain in ridden horses, a Ridden Horse Pain Ethogram (RHpE) was developed, comprising 24 behaviours, the majority of which were at least 10 times more likely to be seen in a lame horse compared with a non-lame horse [5]. Reduction in RHpE behaviour scores, after diagnostic anaesthesia had abolished lameness, verified a causal relationship between the behaviours and pain [6,7]. The display of eight or more behaviours of the RHpE is likely to reflect the presence of musculoskeletal pain [5,6,7,8]. Previous work has focused on comparisons between non-lame horses and horses undergoing veterinary investigation of lameness or poor performance.

Veterinarians, after appropriate training, were able to predict the presence of musculoskeletal pain by application of the RHpE [8]. Other factors which may potentially influence the results of applying the RHpE include an ill-fitting saddle [9], rider size (influenced by height, weight and saddle fit for the rider) [10,11,12] and rider skill [13].

The aim of the current study was to further test the RHpE by its application to a convenience sample of sports horses and riding school horses in regular work and assumed by their owners to be working comfortably. It was hypothesised that there would be a positive association between the RHpE behaviour score and the presence of lameness and/or gait abnormalities in canter.

## 2. Materials and Methods

### 2.1. Data Acquisition

A convenience sample of 64 horse-rider combinations was recruited, comprising volunteers from a further education college (Riding School) and privately owned horses. Horses had to be used to working ‘on the bit’ (with the front of the head in a vertical position), in regular work, and considered by their owners to be non-lame and capable of working for 30 min in trot and canter. Each horse rider combination was unique; no rider rode more than one horse. Age (years), breed (Warmblood, Thoroughbred, Warmblood × Thoroughbred, Cob, Irish Sports Horse or other), sex (mare or gelding) and work discipline (Eventing, Show Jumping, Dressage, General Purpose (including unaffiliated competition), Riding School) were recorded. With the horse standing squarely, before exercise, the thoracolumbosacral region of each horse was palpated by an experienced physiotherapist, using a standardised method. The presence of thoracolumbosacral epaxial muscle hypertonicity (increased muscle tension) and/or pain, subjectively considered to be likely to influence ridden performance, was graded using a binary outcome, yes or no. The saddle of each horse was assessed off the horse and after the rider had tacked up the horse, by a Society of Master Saddlers qualified saddle fitter. The tree stability and soundness, panel and tree symmetry, flocking condition, girth strap alignment and the static fit of the saddle plus pads to the horse were assessed. The fitter was asked to determine whether the tack was likely to induce pain which may compromise performance, graded in binary fashion, yes or no. The riders were advised to use their normal tack; a whip and/or spurs were optional.

All owners gave their informed consent for inclusion prior to participation in the study. The study was conducted in accordance with the Declaration of Helsinki, and the protocol was approved by the Ethics Committees of the Animal Health Trust (AHT 50 2017) and Writtle University (2019). The results for 20 horses have been previously documented [8].

**Figure 1:**
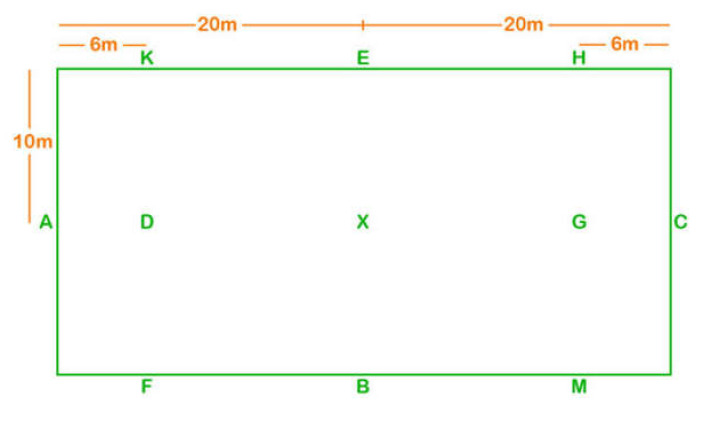
Layout of the dressage arena in which 60 horse rider combinations performed a custom-written dressage test (Table S1). The video recorder stood between M and C, and H and C, for movements 1–9 and 11–26, respectively.

Each horse was ridden by its usual rider. There was a free-exercise warm-up of up to 15 min in an indoor or outdoor arena. Horses which exhibited ≥ grade 3/8 [14] forelimb or hindlimb lameness, as determined by a Diplomate of the European College of Veterinary Sports Medicine and Rehabilitation (SD), during this warm-up were excluded (*n* = 4). Each horse and rider combination then performed a purpose-designed dressage-type test of approximately 8.5 min duration, at walk, trot and canter (Supplementary Materials Table S1 [8]) in a marked 20 × 40 m area (Figure 1), within an indoor arena, at one of three locations. The arena surfaces were sand and rubber. All trot work was performed in rising trot. All 20 m diameter circles were performed at C, close to the video camera; 10 m diameter circles were performed at B and E, respectively. The test, which was of British Dressage Preliminary standard, with the addition of 10 m diameter circles in trot, was called, although the riders had been provided with it in advance.

Video recordings of all tests were acquired from two locations, behind the long sides of the arena, between M and C, and H and C for movements 1–9 and 11–26 (Table S1), respectively. Video recordings were acquired with a high-definition video camera (Panasonic HC-X900, Panasonic Corporation, Hamburg, Germany). Before each test, the size of the iris of each eye and whether or not the sclera could be seen at rest were assessed. Exposure of the sclera at rest and the presence of a bit which was too wide were recorded and referred to when applying the RHpE [5,8].

Throughout the test the gait was assessed subjectively by an experienced lameness diagnostician, used to evaluating ridden horses. The presence of forelimb lameness, hindlimb lameness or lack of hindlimb impulsion and engagement, or an abnormal canter (a stiff and stilted canter, close spatial and/or temporal placement of the hindlimbs, a canter which lacked a suspension phase [15,16], or exaggerated lifting of the forehand during canter, associated with abnormally wide spatial separation of the hindlimbs [17]) was recorded. Lameness was graded on a 0–8 scale [14].

The RHpE (Table 1) [5] was applied retrospectively to the video recordings by a trained assessor, without knowledge of the live gait assessment results. The video recordings were also assessed independently by a British Horse Society Instructor, who graded the skill of the rider on the Fédération Equestre Internationale 1–10 scale (Supplementary Materials Table S2) [18] based on application of the aids, balance, position and core stability. This assessor was blinded to all other results, the experience of the riders and the work discipline of the horses.

All riders were provided with both verbal and written feedback about the results of palpation of the thoracolumbosacral region, saddle-fit and gait abnormalities after completion of their tests.

### 2.2. Data Analysis

Data were collated into a Microsoft Excel (Office 365) spreadsheet (Microsoft Corporation, Redmond, Washington, USA) and imported into statistical software Stata (IC v.13.0, StataCorp LP, College Station, TX, USA) for coding and analyses.

### 2.3. Descriptive Statistics

Normality of distribution for continuous variables was visually assessed with histograms and overlaid normal and kernel density plots. Continuous and ordinal variables were described as medians with corresponding ranges and interquartile ranges (IQR), and categorical variables were described as proportions with 95% confidence intervals (CI).

### 2.4. Initial Associations

The prevalence of each of the 24 RHpE behaviours was compared between lame and non-lame horses with a Fisher’s exact test. Differences in RHpE scores across categorical variables were initially assessed using the Mann–Whitney *U* test for variables with two categories, and the Kruskal–Wallis test for variables with more than two categories. The Spearman rank correlation coefficient was used to assess correlations between RHpE score and horse age and rider skill score. Significance was set at *p* < 0.05 and *p*-values were adjusted for multiple comparisons using the Benjamini–Hochberg false discovery rate methods, with a false discovery rate of 0.05 [19]. Both unadjusted and adjusted *p*-values are presented.

### 2.5. Poisson Regression

Univariable Poisson regression, with robust standard errors, was used to calculate incident rate ratios (IRR) and corresponding 95% CIs in order to identify factors associated with higher RHpE scores [20]. The use of robust standard errors is recommended in Poisson regression in order to control for mild violations of the underlying assumptions, most notably the assumption that the mean and variance of the outcome variable distribution are equal [20]. Age was additionally recoded into quartile categories in order to test for evidence against linearity. Variables where Wald *p* < 0.25 were taken forward to multivariable Poisson regression modelling. Manual, forward selection was used to create the final multivariable Poisson regression model, with robust standard errors, and variables were added to the model in a stepwise manner from most to least significant based on their Wald *p*-values. Variables were retained in the final model where Wald *p* < 0.05 and if they significantly improved model fit in a model without robust standard errors (likelihood ratio statistic *p* < 0.05). All variables not retained in the final model were individually forced back into the model in order to not omit any interactions or confounders. The goodness-of-fit chi-squared test was used to assess the fit of the model to the data.

## 3. Results

### 3.1. Descriptive Statistics

The median age of the horses was 11 years (IQR 9–14; range 5–24 years). Breeds were comprised of 16 Warmbloods (26.7%, CI 15.5, 37.9), 13 Irish Sports Horses (21.7%, CI 11.2, 32.1), nine Thoroughbreds (15.0%, CI 6.0, 24.0%), eight Cobs (13.3%, CI 4.7, 21.9%), six Warmblood × Thoroughbreds (10.0%, CI 2.4, 17.6%) and eight other breeds (13.3%, CI 4.7, 21.9%) including three Connemara ponies, two Welsh ponies, one Andalusian, one Irish Draught and one Shire. The majority of the horses were geldings (*n* = 36; 60%, CI 47.6, 72.4%) rather than mares (*n* = 24; 40%, CI 27.6, 52.4%). The most common work discipline was General Purpose (*n* = 26; 43.3%, CI 30.8, 55.9%), followed by Dressage (*n* = 12; 20.0%, CI 9.9, 30.1%) and Riding School (*n* = 11; 18.3%, CI 8.5, 28.1%), with the least number of horses in the Eventing (*n* = 8) and Show Jumping (*n* = 3) disciplines. Lameness was recognised in 73.3% (CI 62.1, 84.5; *n* = 44) of the horses, while 46.7% (CI 34.0, 59.3%; *n* = 28) had abnormalities in canter. Twenty-two (36.7%, CI 24.5, 48.9%) horses were both lame and had an abnormal canter. Increased tension or pain in the thoracolumbosacral epaxial muscles was identified in 58.3% (CI 45.9, 70.8%; *n* = 35) of the horses, while 46.7% (CI 34.0, 59.3%; *n* = 28) were found to have saddle fit which could negatively affect performance. The median rider skill score was 4.75 (IQR 4–5.5; range 2.5–8). The median RHpE score out of 24 was 9.0 (IQR 7–11; range 3–16).

### 3.2. Initial Associations

When considering the prevalence of RHpE behaviours in lame and non-lame horses, in the unadjusted analysis lame horses were more likely to flatten or rotate their ears backwards (*p* = 0.03), exhibit an intense stare (*p* = 0.02) and stumble or trip repeatedly (*p* = 0.04) compared with non-lame horses (Table 2). However, the significance of these findings was not retained following adjusting for multiple comparisons. Results of the unadjusted Mann–Whitney *U* and Kruskal–Wallis tests indicated that higher RHpE scores were associated with lameness (*p* = 0.02) only. However, potential associations of interest (*p* < 0.10) additionally included sex (*p* = 0.07) and work discipline (*p* = 0.07) (Table S3). Mares had higher RHpE scores compared with geldings, and Riding School horses had the highest median RHpE scores from all the work disciplines. Following adjustment for multiple comparisons, only lameness (*p* = 0.18) and work discipline (*p* = 0.21) retained an adequate level of significance in relation to the *p* < 0.25 cut-off used for screening variables for inclusion in multivariable analyses. A correlation between horse age and RHpE score was not identified (Spearman’s rho = 0.04, *p* = 0.77). Rider skill score had a negative correlation with RHpE score (Spearman’s rho = −0.45, *p* = 0.0003, adjusted *p* = 0.0006).

### 3.3. Poisson Regression

Univariable Poisson regression analysis identified six variables to take through to multivariable modelling (Table 3). These included rider skill score (*p* < 0.001), work discipline (*p* < 0.001), lameness (*p* = 0.03), both lameness and abnormal canter (*p* = 0.08), sex (*p* = 0.09), abnormal canter (*p* = 0.12) and age (*p* = 0.17). The results of the univariable Poisson regression model complement the findings of the initial pairwise comparisons, with lameness status and work discipline identified as variables with the strongest relationship with RHpE score. Similarly, correlation analysis had identified a strong negative linear relationship between RHpE score and rider skill score, which was replicated in the regression analysis, helping describe how rider skill score was numerically related to RHpE score.

**Figure 2:**
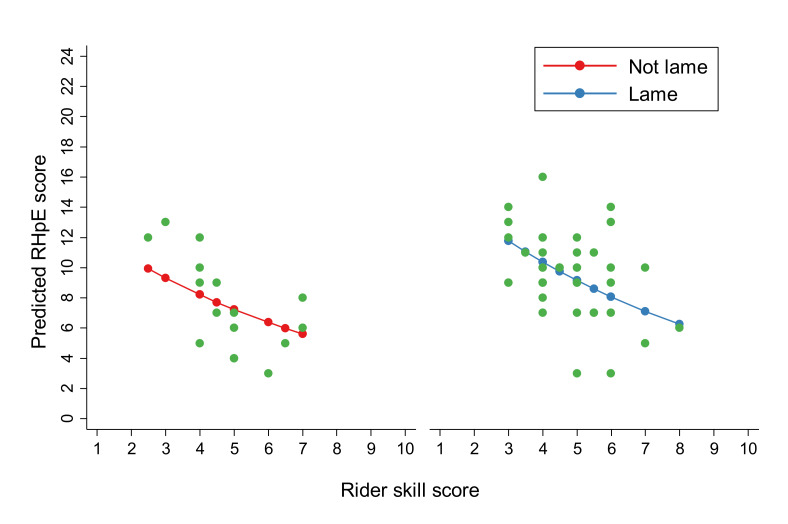
Prediction of linear Ridden Horse Pain Ethogram (RHpE) scores based on rider skill score (0–10) and lameness status (red = non-lame; blue = lame), as estimated by a multivariable Poisson regression model with robust standard errors. Individual points (green) represent the original data used to make these linear predictions.

**Figure 3:**
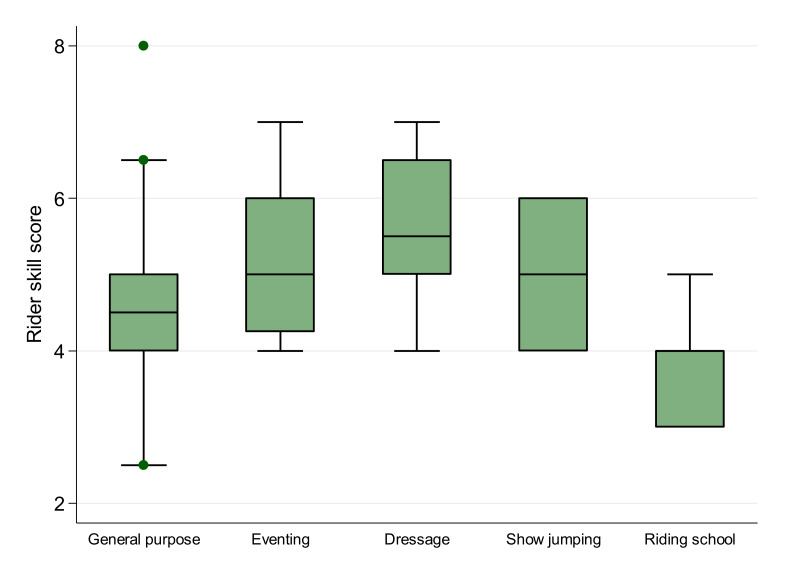
Median rider skill scores (0–10) of 60 riders across five equestrian work disciplines. Boxes represent medians and interquartile ranges, whiskers represent the range and individual points outliers.

The final multivariable Poisson regression model identified associations between RHpE score and both rider skill score and lameness (Table 4). The change in the incident rate of RHpE scores decreased by 0.88 for every unit increase in rider skill score (*p* < 0.001). The incident rate of RHpE scores for lame horses was 1.26 times higher than for non-lame horses (*p* = 0.008). The predicted RHpE score from the model, given rider skill score and lameness status, is presented in Figure 2. The goodness-of-fit chi-squared test indicated the Poisson model was an adequate fit for the data (goodness-of-fit χ^2^ = 41.8, *p* = 0.93). Although work discipline was not retained in the final model, this is likely because there is a relationship between rider skill score and work discipline, with riders of Riding School horses having the lowest median rider skill score from all the work disciplines (Kruskal–Wallis *p* = 0.001) (Figure 3). A difference in lameness across the work disciplines was not identified (*p* = 0.43).

The results of the multivariable analysis clearly show a strong positive relationship between the presence of lameness and the RHpE score. All non-lame horses that had a normal canter had a RHpE score <8 (median 5, range 3–6), with the exception of one Riding School horse and one General Purpose horse. Moreover, 10 of 11 Riding School horses had gait abnormalities consistent with musculoskeletal pain and Riding School horses had the highest median RHpE score from all work disciplines. A RHpE score ≥8 is likely to reflect the presence of musculoskeletal pain.

## 4. Discussion

In accordance with our hypothesis there was a positive association between RHpE scores and the presence of lameness and, given the results of previous studies [6,7,8], this is likely to be a causal relationship. However, contrary to our hypothesis, gait abnormalities in canter, either alone or in combination with lameness, although carried forward to the multivariable analysis, were not significant in the final model. In addition, rider skill score was negatively associated with the RHpE score. However, this may not be a causal relationship. Although work discipline was not retained in the final model, this is probably because riders of the Riding School horses, which had the highest median RHpE score, had the lowest median rider skill score from all the work disciplines.

There are several studies which demonstrate that unskilled novice riders have a less stable position and lack of phase synchrony with the horse compared with riders of superior skill and experience [21,22,23,24]. These features have the potential to alter the horse’s head and neck position [25], lameness [26], and quality of gaits [27]. However, we have previously observed during clinical investigation of lame horses that, when ridden by a skilled professional, the rider may appear to ride relatively poorly. However, when gait abnormalities are abolished by local anaesthesia, the rider then appears to be both more in harmony with the horse and considerably more proficient. Moreover, 40 of the horses in the current study were part of a related study in which all horses were ridden by the normal rider and a professional rider [28]. The professional rider’s skill score ranged from 3.5 to 7/10 (median 6), being strongly influenced by the presence (low rider skill score) or absence (high rider skill score) of lameness [28]. In a study involving eight riders, four beginners and four more experienced riders, and eight riding school horses there was no effect of rider skill on a limited number of horse behaviours [13]. As might be expected, in the current study rider skill level was higher for horses used for affiliated level competition compared with General Purpose and Riding School horses, but overall the median score was low (4.75/10), corresponding to less than sufficient as defined by the Fédération Equestre Internationale [18]. It remains possible that an unskilled rider riding a pain-free horse may alter its behaviour.

There was a high frequency of low-grade (≤ grade 2/8 [14]) lameness (73%) and other gait abnormalities (47%) in the current study, reflecting owners’ inability to recognise musculoskeletal pain, as previously documented [3,4,29]. It should be borne in mind that in horses with lameness in more than one limb, as frequently observed in the current study, the grading of lameness may be neither accurate nor reflect the level of discomfort [14]. Education of riders and trainers about the value of the RHpE for detecting the likely presence of musculoskeletal pain may prompt them to seek professional advice. Lameness was not necessarily a continuous feature, but was often accentuated in 10 m diameter circles in trot, highlighting the value of this exercise when investigating poor performance and at pre-purchase examinations. The test performed by the riders was purpose-designed to include 10 m diameter circles because it has previously been observed that this movement can influence both lameness and behaviour [6]. This study also reinforces the importance of evaluating the quality of canter, which may be compromised especially in association with hindlimb lameness or sacroiliac joint region pain [17,30,31,32]. Although gait abnormalities in canter were not significant in the final model, it does not mean that such abnormalities are not of biological significance. It has previously been observed, in a minority of horses, that no behavioural or gait abnormalities were detectable in trot, whereas gait abnormalities in canter were seen in association with a high RHpE score [6].

Of the Riding School horses 9/11 (82%) showed lameness and 6/11 (55%) displayed gait abnormalities in canter; only one horse was graded as displaying no gait abnormalities, but this horse worked consistently with the front of the head in front of the vertical > 30° ≥10 s and had reduced range of motion of the thoracolumbosacral region, and had a RHpE score of 13. Overall, the median RHpE score for the Riding School horses was higher than for the other work disciplines. There are limited published data concerning riding school horses. In a Swedish study of 99 horses from eight riding schools, 15 exhibited ‘moderate lameness’ in hand or on the lunge, including eight with gait abnormalities in canter [33]. However, ‘irregular movement’ (presumably low-grade lameness) was observed in an additional 45 limbs and a further 17 horses were described as ‘moving short’ and nine ‘moved flat on the ground’. Thus, potentially up to 87% of the horses had gait abnormalities reflecting musculoskeletal pain. In a cross-sectional study of 2566 Dutch riding horses from 150 horse farms, 19.3% were lame or had ‘irregular locomotion’ when assessed moving in hand by trained veterinary students [34]. Riding school horses had a higher risk of lameness than horses used for recreation or competition. The sample size of Riding School horses in the current study was small, but if it is representative of the United Kingdom Riding School population, the high incidence of lameness and RHpE scores ≥8 is of cause for concern. Under the Riding Establishments Act (1964) horses have to undergo a statutory annual veterinary inspection to determine that they are ‘in all aspects physically fit’. This examination does not require horses to be ridden. Education of riding school proprietors about the RHpE and the use of ridden exercise and application of the RHpE by veterinarians during their inspections may enhance riding school horse welfare.

The RHpE scores ranged from 3 to 16/24, with a median of 9. These scores were substantially higher than those documented at three 5* three-day events during warm-up for the dressage phase, at which the scores of 172 horses ranged from 0 to 9 (median 4) [35,36]. In the latter studies, horses with a score ≥7/24 performed significantly less well in the dressage and cross-country phases and had lower overall places, compared with horses that scored <7. In the current study, with the exception of one Riding School horse and one General Purpose horse, all non-lame horses that had a normal canter had a RHpE score <8 (range 3–6, median 5), consistent with previous reports [5,6,7]. This reinforces that a RHpE score ≥8 is likely to reflect musculoskeletal pain. We have previously demonstrated through assessment of sensitivity and specificity that a threshold of 8 is appropriate for the differentiation of horses with and without musculoskeletal pain [7]. It was clear that riders, irrespective of their skill level and experience, had a lack of recognition of the behavioural signs which may reflect musculoskeletal pain. We believe that many riders learn to ride on riding school horses which exhibit these signs, so that there is a generalised acceptance that these behaviours are normal for horses. There is an urgent need for education of riders and trainers at all levels to recognise that demonstration of eight or more behaviours of the RHpE is highly likely to reflect musculoskeletal pain.

Overall, the incident rate of each of the RHpE scores for lame horses was 1.27 times the incident rate for the non-lame horses. Those features which had the strongest relationship with lameness were ears back for ≥ 5 s, an intense stare for ≥ 5 s, and repeated stumbling or bilateral hindlimb toe drag, similar to previous observations [7]. Although these associations were not retained following adjustment for multiple comparisons, this likely reflects the limitations of the sample size and the variation with which individual horses show pain-related behaviours. As previously documented [5,6] and as illustrated in Table 2, different horses show different behaviour patterns and therefore reliance on a limited spectrum of behaviours is potentially misleading.

In the current study the presence of thoracolumbosacral epaxial muscle pain or increased muscle tension did not influence the RHpE score. Although palpation is a subjective assessment, there is evidence that it may be more reliable than more objective assessments [37]. There is a relationship between the presence of either lameness [38] or sacroiliac joint region pain [32] and epaxial muscle hypertonicity and pain. We have observed that when pain associated with lameness or sacroiliac joint region pain is abolished using local anaesthesia, there is a substantial reduction in the RHpE scores, despite the persistence of epaxial muscle hypertonicity or pain [6,7], which is consistent with the results of the current study.

There was a disturbingly high proportion (47%) of ill-fitting saddles with the potential to adversely influence performance. However, in the current study, saddle fit did not influence the RHpE score. Nonetheless, a change from an ill-fitting saddle to a better fitting saddle may have a dramatic influence on ridden horse behaviour [39]. Twenty-one horses were assessed ridden by a single professional rider in both a well-fitting saddle and an ill-fitting saddle [40]. There was significantly less variability in gait with the better-fitting saddle compared with the ill-fitting saddle. It is therefore considered important to address saddle fit when assessing a horse with poor performance. If a high RHpE score persists after abolition of lameness using diagnostic anaesthesia this usually indicates the presence of an additional problem, such as an ill-fitting saddle, meriting further investigation [7,41].

A small proportion (3%) of horses that had a RHpE score of ≥8 did not show overt lameness or gait abnormalities, as defined, in canter. There are other gait variants which are likely to reflect adaptations to musculoskeletal pain which are not covered by the conventional understanding of lameness, the definitions employed for this study, or behaviours within the ethogram. These include a short stepping forelimb gait; reduced range of motion of the thoracolumbosacral region; reduced flexion of the lumbosacral joint; ‘on the forehand’; scooting forwards episodically; ‘jumping’ into transitions to trot from walk; transiently going above the bit in upward transitions; stepping short on the hindlimbs in downward transitions from canter to trot, or from trot to walk; ‘croup high’ in canter; and altered movement of the thoracolumbar region in canter resulting in rotation of the rider’s pelvis [15,16,30,32,42]. It is therefore suggested that it is crucial to apply the RHpE to horses performing both trot and canter and also to observe behaviour and movement quality in transitions between gaits.

An inherent limitation of the study design is that the trained assessor who applied the RHpE could not be blinded to the lameness status of the horse, work quality or the skill of the rider. Assessment of epaxial muscle tension or pain, saddle fit, lameness and canter, and rider skill were all subjective, but were performed by independent experts, with no knowledge of the RHpE scores. Quantitative assessment of ridden horse gait [43] has many limitations, especially in a horse lame on more than one limb, and for lameness that varies between reins and different movements, and cannot be used to assess transitions between gaits or canter. The gait may alter between rising and sitting trot and vary depending on the diagonal on which the rider sits [15,43], however, in the current study all trot work was performed in rising trot, because the majority of the riders rarely rode in sitting trot. Riders sat on the correct diagonal, when the outside forelimb and inside hindlimb were bearing weight. There are other potential confounders that were not identified or measured in the current study. For example, although bit fit was assessed, the mouth was not inspected, so the presence of oral lesions that could have contributed to discomfort cannot be excluded. Noseband type and fit were recorded, but not included in the analyses. Poisson regression generally requires a large sample size, so it is likely that our model did not have sufficient power to detect more subtle associations. However, the results of the Poisson regression reflected the results of the pairwise comparisons and allowed us to fully utilise the data and to assess the effect of multiple variables on RHpE scores in the same model. The study population was a convenience sample and may not be representative of the United Kingdom horse population as a whole.

There has been limited comparison between physiological markers of stress and ridden horse behaviour [44]. In a previous review of ridden horse behaviour, the difficulties of differentiation between behaviour reflecting pain, anxiety, fear and mental state were highlighted, together with the potential overlap between physiological signs of stress and the response to physical exertion [45]. The RHpE appears to provide a simple method of determining the likely presence of musculoskeletal pain, with the current study providing further evidence of the strong association between a RHpE score ≥8 and the presence of lameness.

## 5. Conclusions

There was a negative association between rider skill score and the RHpE score, but this is not believed to be a causal relationship. There was a positive association between lameness and the RHpE score, and this provides further evidence that a score of ≥8/24 reflects the presence of musculoskeletal pain. A relationship between RHpE score and work discipline was not found to be significant after adjustment for lameness and rider skill score, although Riding School horses had the highest median RHpE scores out of all the other work disciplines. This is likely because of a strong relationship between work discipline and rider skill score, with riders of riding school horses having lowest median rider skill scores. Behaviours exhibited by many riding school horses are likely to reflect chronic discomfort and should not be regarded as typical behaviour of pain-free horses. Education of owners, riders, trainers and all associated professionals is required about what constitutes abnormal horse behaviour, so that lame horses are recognised and undergo appropriate investigation and treatment, in order to both enhance equine welfare and improve performance.

## Figures and Tables

**Table 1:** The Ridden Horse Pain Ethogram, adapted from Dyson et al. 2018 [5]. Assessments were made in walk, trot and canter and on the left and right reins. A total behaviour score of ≥8 (out of 24) is likely to indicate the presence of musculoskeletal pain (Dyson et al. 2018 [5,6]). s = seconds.

1. Repeated changes of head position (up/down), not in rhythm with the trot
2. Head tilted or tilting repeatedly
3. Head in front of vertical (>30°) for ≥10 s
4. Head behind vertical (>10°) for ≥10 s
5. Head position changes regularly, tossed or twisted from side to side, corrected constantly
6. Ears rotated back behind vertical or flat (both or one only) ≥5 s; repeatedly lay flat
7. Eye lids closed or half closed for 2–5 s; frequent blinking
8. Sclera exposed repeatedly
9. Intense stare (glazed expression, ‘zoned out’) for ≥5 s
10. Mouth opening ± shutting repeatedly with separation of teeth, for ≥10 s
11. Tongue exposed, protruding or hanging out, and/or moving in and out repeatedly
12. Bit pulled through the mouth on one side (left or right), repeatedly
13. Tail clamped tightly to middle or held to one side
14. Tail swishing large movements: repeatedly up and down/side to side/circular; repeatedly during transitions
15. A rushed gait (frequency of trot steps > 40/15 s); irregular rhythm in trot or canter; repeated changes of speed in trot or canter
16. Gait too slow (frequency of trot steps < 35/15 s); passage-like trot
17. Hindlimbs do not follow tracks of forelimbs but repeatedly deviated to left or right; on three tracks in trot or canter
18. Canter repeated leg changes in front and/or behind; repeated strike off on wrong leg; disunited
19. Spontaneous changes of gait (e.g., breaks from canter to trot, or trot to canter)
20. Stumbles or trips more than once; repeated bilateral hindlimb toe drag
21. Sudden change of direction, against rider’s direction; spooking
22. Reluctance to move forwards (has to be kicked ± verbal encouragement), stops spontaneously
23. Rearing (both forelimbs off the ground)
24. Bucking or kicking backwards (one or both hindlimbs)

**Table 2:** The prevalence, difference in prevalence and Fisher’s exact *p*-value of 24 Ridden Horse Pain Ethogram behaviours compared between lame and non-lame ridden horses (*n* = 60).

Ridden Horse Pain Ethogram Behaviour	Prevalence in Lame Horses (*n* = 44)	Prevalence in Non-Lame Horses (*n* = 16)	Difference in Prevalence (%)	Fisher’s Exact *p*-Value (Benjamini–Hochberg Adjusted *p*-Value ^*^)
1. Repeated changes of head position (up/down)	24 (54.5%)	9 (56.3%)	−1.8%	1.00 (1.00)
2. Head tilted or tilting repeatedly	33 (75.0%)	12 (75.0%)	+0.0%	1.00 (1.00)
3. Head in front of vertical (>30°) for ≥10 s	23 (52.3%)	5 (31.3%)	+21.0%	0.24 (1.00)
4. Head behind vertical for >10 s	17 (38.6%)	5 (31.3%)	+7.3%	0.76 (1.00)
5. Head position changes regularly, tossed or twisted from side to side, corrected constantly	15 (34.1%)	8 (50.0%)	−15.9%	0.37 (0.85)
6. Ears rotated back behind vertical or flat (both or one only) ≥5 s; repeatedly lay flat	34 (77.3%)	7 (43.8%)	+33.5%	**0.03** (0.35)
7. Eye lids closed or half closed for 2–5 s; repeated	5 (11.4%)	1 (6.3%)	+5.1%	1.00 (1.00)
8. Sclera (white of eye) exposed	17 (38.6%)	6 (37.5%)	+1.1%	1.00 (1.00)
9. Intense stare for ≥5 s	36 (81.8%)	8 (50.0%)	+31.8%	**0.02** (0.46)
10. Mouth opening ± shutting repeatedly with separation of teeth, for ≥10 s	27 (61.4%)	7 (43.8%)	+17.6%	0.25 (0.96)
11. Tongue exposed, protruding or hanging out and/or moving in and out	18 (40.9%)	4 (25.0%)	+15.9%	0.37 (0.77)
12. Bit pulled through the mouth on one side (left or right)	13 (29.6%)	3 (18.8%)	+10.8%	0.52 (0.92)
13. Tail clamped tightly to middle or held to one side	11 (25.0%)	4 (25.0%)	+0.0%	1.00 (1.00)
14. Tail swishing large movements: repeatedly up and down/side to side/circular; during transitions	23 (52.3%)	6 (37.5%)	+14.8%	0.39 (0.75)
15. A rushed gait (frequency of trot steps > 40/15 s); irregular rhythm in trot or canter; repeated changes of speed in trot or canter	6 (13.6%)	2 (12.5%)	+1.1%	1.00 (1.00)
16. Gait too slow (frequency of trot steps <35/15 s); passage-like trot	4 (9.1%)	0 (0.0%)	+9.1%	0.57 (0.94)
17. Hindlimbs do not follow tracks of forelimbs but deviated to left or right; on 3 tracks in trot or canter	36 (81.8%)	13 (81.3%)	+0.5%	1.00 (1.00)
18. Canter repeated leg changes: repeated strike off wrong leg; change of leg in front and/or behind; disunited	4 (9.1%)	3 (18.8%)	−9.7%	0.37 (1.00)
19. Spontaneous changes of gait (e.g., breaks from canter to trot or trot to canter)	28 (63.6%)	7 (43.8%)	+19.8%	0.24 (1.00)
20. Stumbles or trips/catches toe repeatedly	28 (63.6%)	5 (31.3%)	+32.3%	**0.04** (0.31)
21. Sudden change of direction, against rider direction; spooking	4 (9.1%)	3 (18.8%)	−9.7%	0.37 (0.95)
22. Reluctant to move forward (has to be kicked ± verbal encouragement), stops spontaneously	13 (29.6%)	2 (12.5%)	+17.1%	0.31 (1.00)
23. Rearing (both forelimbs off the ground)	0 (0.0%)	0 (0.0%)	+0.0%	-
24. Bucking or kicking backwards (one or both hindlimbs)	1 (2.3%)	0 (0.0%)	+2.3%	1.00 (1.00)

^*^ False discovery rate *p*-value of 0.05 to adjust for multiple comparisons; bold values indicate significance at *p* < 0.05.

**Table 3:** Univariable Poisson regression, including robust standard errors, of signalment, work discipline and lameness-related variables associated with Ridden Horse Pain Ethogram scores in a convenience sample of 60 horses. CI = confidence intervals.

Variable	Coefficient	Robust Standard Error	Incident Rate Ratio (IRR)	IRR 95% CI	Wald *p*-Value
*Age (continuous)*	0.02	0.01	1.02	0.99, 1.04	**0.165**
*Age quartiles*					**0.185**
5–9 years	Reference		Reference		
10–11 years	−0.02	0.10	0.98	0.81, 1.18	0.83
12–14 years	−0.16	0.12	0.86	0.68, 1.07	0.18
15–24 years	0.12	0.11	1.13	0.91, 1.41	0.28
*Breed*					0.28
Warmblood	Reference		Reference		
Irish Sports Horse	−0.06	0.21	0.94	0.63, 1.42	0.18
Thoroughbred	0.17	0.14	1.19	0.90, 1.57	0.22
Cob	0.19	0.11	1.21	0.98, 1.50	0.08
Warmblood × Thoroughbred	0.17	0.13	1.18	0.93, 1.51	0.79
Other	0.28	0.13	1.32	1.02, 1.72	0.04
*Sex*					**0.090**
Mare	0.12	0.08	1.15	0.98, 1.34	
Gelding	Reference		Reference		
*Work discipline*					**<0.001**
General purpose	Reference		Reference		
Dressage	0.01	0.12	1.01	0.79, 1.29	0.94
Riding School	0.24	0.08	1.27	1.08, 1.50	0.004
Eventing	0.08	0.12	1.09	0.86, 1.38	0.48
Show Jumping	−0.10	0.10	0.90	0.74, 1.10	0.32
*Lame*					**0.027**
Yes	0.24	0.11	1.27	1.03, 1.58	
No	Reference		Reference		
*Abnormal canter*					**0.119**
Yes	0.13	0.08	1.13	0.97, 1.33	
No	Reference		Reference		
*Both lame and abnormal canter*					**0.083**
Yes	0.13	0.08	1.14	0.98, 1.33	
No	Reference		Reference		
*Pain or increased tension in the thoracolumbosacral epaxial muscles*					0.389
Yes	−0.07	0.08	0.93	0.80, 1.09	
No	Reference		Reference		
*Saddle fit likely to negatively affect performance*					0.530
Yes	−0.05	0.08	0.95	0.80, 1.12	
No	Reference		Reference		
*Rider skill score (continuous)*	*−0.13*	*0.03*	*0.88*	*0.83, 0.94*	**<0.001**

Bold values indicate significance at *p* < 0.25.

**Table 4:** The final multivariable Poisson regression, including robust standard errors, of signalment, work discipline and lameness-related variables associated with Ridden Horse Pain Ethogram scores in a convenience sample of 60 horses. CI = confidence intervals.

Variable	Coefficient	Robust Standard Error	Incident Rate Ratio (IRR)	IRR 95% CI	Wald *p*-Value
*Rider skill score (continuous)*	−0.13	0.03	0.88	0.83, 0.94	**<0.001**
*Lame*					**0.008**
Yes	0.23	0.09	1.26	1.06, 1.50	
No	Reference		Reference		

## Supplementary Materials

The following are available online at https://www.mdpi.com/2076-2615/10/6/1044/s1.
